# High-Pressure Carbon Monoxide and Oxygen Mixture is Effective for Lung Preservation

**DOI:** 10.3390/ijms20112719

**Published:** 2019-06-03

**Authors:** Atsushi Fujiwara, Naoyuki Hatayama, Natsumi Matsuura, Naoya Yokota, Kaori Fukushige, Tomiko Yakura, Shintaro Tarumi, Tetsuhiko Go, Shuichi Hirai, Munekazu Naito, Hiroyasu Yokomise

**Affiliations:** 1 Department of General Thoracic, Breast and Endocrinological Surgery, Kagawa University, Kagawa 761-0793, Japan; a24fujiwara@gmail.com (A.F.); nmori1130@gmail.com (N.M.); naoe@med.kagawa-u.ac.jp (N.Y.); shintarotarumi@gmail.com (S.T.); g-tetsu@wa3.so-net.ne.jp (T.G.); yokomise@med.kagawa-u.ac.jp (H.Y.); 2 Department of Anatomy, Aichi Medical University, Aichi 480-1195, Japan; nhatayama416@gmail.com (N.H.); kaori.fukushige@gmail.com (K.F.); tomi.tomi105@gmail.com (T.Y.); munekazunaito@gmail.com (M.N.)

**Keywords:** carbon monoxide, lung preservation, ischemia–reperfusion injury, high-pressure gas

## Abstract

(1) Background: Heme oxygenase-1 (HO-1) degrades heme and generates carbon monoxide (CO), producing various anti-inflammatory, anti-oxidative, and anti-apoptotic effects. This study aimed to confirm the effects of CO on the ischemia–reperfusion injury (IRI) of donor lungs using a high-pressure gas (HPG) preservation method. (2) Methods: Donor rat and canine lungs were preserved in a chamber filled with CO (1.5 atm) and oxygen (O_2_; 2 atm) and were ventilated with either CO and O_2_ mixture (CO/O_2_ group) or air (air group) immediately before storage. Rat lungs were subjected to heterotopic cervical transplantation and evaluated after reperfusion, whereas canine lungs were subjected to allogeneic transplantation and evaluated. (3) Results: Alveolar hemorrhage in the CO/O_2_ group was significantly milder than that in the air group. mRNA expression levels of HO-1 remained unchanged in both the groups; however, inflammatory mediator levels were significantly lower in the CO/O_2_ group than in the air group. The oxygenation of graft lungs was comparable between the two groups, but lactic acid level tended to be higher in the air group. (4) Conclusions: The HO-1/CO system in the HPG preservation method is effective in suppressing IRI and preserving donor lungs.

## 1. Introduction

Carbon monoxide (CO), a colorless and odorless gas, produces carboxyhemoglobin (CO-Hb) and inhibits the P450 enzyme of the mitochondrial electron transfer system [1,2], resulting in tissue oxygen deficiency. Moreover, endogenous CO is biosynthesized when heme oxygenase-1 (HO-1) is involved in the degradation of hemoglobin to bilirubin [3]. CO plays anti-inflammatory, anti-oxidative, vasodilatory, and anti-apoptotic roles. The p38 mitogen-activated protein kinase pathway is reportedly one of the mechanisms underlying the cytoprotective effects of CO [4,5,6,7]. Furthermore, exogeneous CO administration therapeutically affects transplantation by suppressing ischemia–reperfusion injury (IRI) [8,9,10]. Therefore, the HO-1/CO system has protective effects against stress reactions.

Lung transplantation is an important treatment approach for end-stage lung disease. However, ischemia–reperfusion injury (IRI), which is one of the most important complications of lung transplantation, hinders its utility and efficacy [11]. Mortality rates after transplantation are higher in cases with severe IRI, which is difficult to control. Successful IRI suppression is critical for maintaining good preservation conditions. Although the simple immersion method using an organ preservation solution is the globally used and clinically applied approach for lung preservation during transplantation, it cannot completely prevent damage due to IRI [12,13,14]. To suppress IRI in donor lungs, several recent methods, including ex vivo lung perfusion, have been explored, and the development of new organ preservation methods that can satisfactorily preserve lungs for extended periods is urgent.

We recently developed a high-pressure gas (HPG) preservation method using a mixture of CO and oxygen (O_2_) and succeeded in resuscitating rat hearts after long-term preservation [15,16], revealing the utility of this method through histological analysis and metabolic evaluation [17]. Furthermore, this preservation method was reportedly useful for the preservation of rat kidneys [18] and limbs [19]. In addition, the lung is a solid organ similar to the kidney and liver, although it can be filled with gas from the inside, which is its decisively unique character. Appropriate delivery of CO and O_2_ in their gaseous forms to the alveoli, in addition to their simple diffusion from the serosal side in a hyperbaric chamber, is anticipated to maximize their organ-preserving effects. Therefore, we hypothesized that the lung is a suitable organ to test the advantages of the HO-1/CO system in the HPG preservation method and investigated the utility of this approach using rat and canine models of lung transplantation.

## 2. Results

### 2.1. Experiment 1. Comparison of the Preservation Status of Rat Donor Lungs Filled and Preserved with Mixture of CO and O_2_ or Air

**Figure 1:**
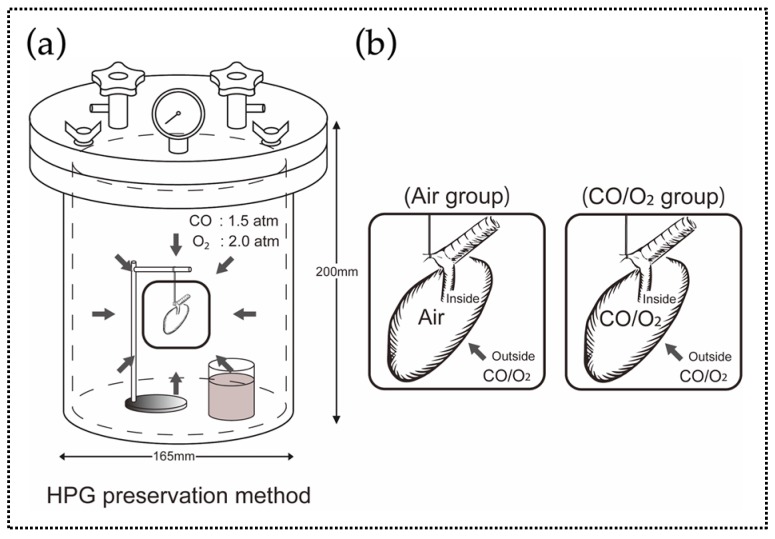
Schematic representation of the preservation method (Experiment 1). (**a**) Donor lungs were preserved in a chamber with high-pressure carbon monoxide (CO; 1.5 atm) and oxygen (O_2_; 2.0 atm). (**b**) Before preservation, donor lungs were filled with either room air (containing nitrogen (N_2_) and O_2_ at a ratio of 4:1; air group) or a mixture of CO and O_2_ at the same ratio as that outside (CO:O_2_, 3:4; CO/O_2_ group).

We evaluated the preservation status of rat donor lungs preserved using the HPG preservation method. The donor lungs were filled with a mixture of CO and O_2_ or air (Figure 1).

**Figure 2:**
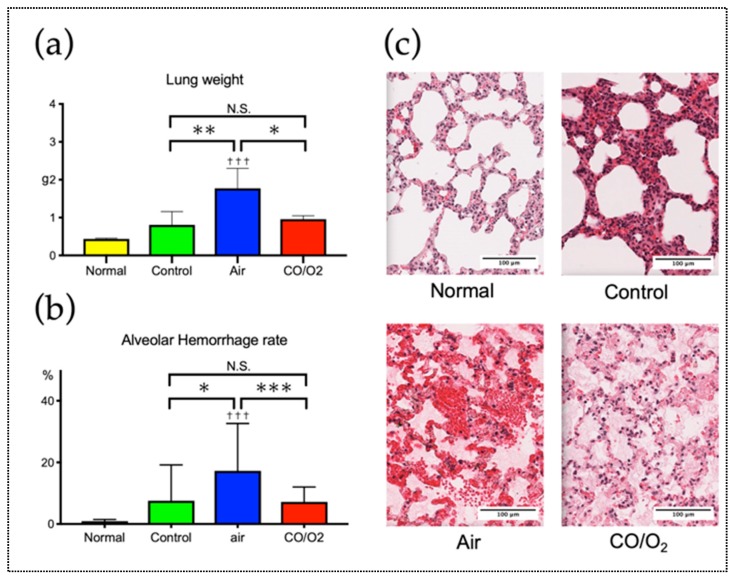
Assessment of rat lungs in the 24-h preservation model. (**a**) Comparison of the lung weights among the four groups. (**b**) Comparison of the severity of alveolar hemorrhage determined by histological assessment among the four groups. (**c**) Comparison of changes observed by light microscopy among the four groups. Each data of bars are expressed as the means ± standard deviations. N.S.—not significant, *** *p* < 0.001, ** *p* < 0.01, * *p* < 0.05, ^†††^ *p* < 0.001 compared with the normal group. Black bar = 100 μm

Mean weight of the donor lungs in the normal group (0.44 ± 0.01 g) was not significantly different from that of the donor lungs in the control (0.81 ± 0.35 g, *p* = 0.509) and CO/O_2_ (0.97 ± 0.09 g, *p* = 0.212) groups. Mean weight of the donor lungs of the air group (1.77 ± 0.53 g) was significantly greater than that of the donor lungs in the normal (*p* < 0.001), control (*p* = 0.005), and CO/O_2_ groups (*p* = 0.011; Figure 2a). Light microscopic evaluation of alveolar hemorrhage, which is an indicator of IRI, revealed that similar to the result observed in terms of lung weight, there was no significant difference between the control and CO/O_2_ groups (*p* = 0.999). However, there was significantly more severe alveolar hemorrhage in the air group than in the CO/O_2_ group (*p* = 0.005). A similar trend was observed among the air, control, and CO/O_2_ groups (Figure 2b,c).

**Figure 3:**
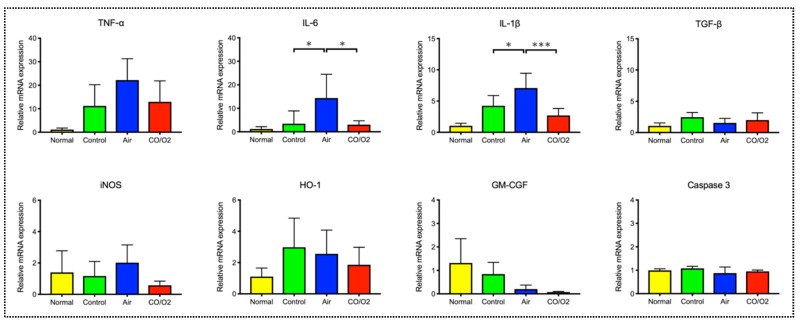
Changes in relative mRNA expression levels of mediators in donor rat lungs after 90 min of reperfusion. Each data of bars are expressed as the means ± standard deviations. *** *p* < 0.001, * *p* < 0.05.

Real-time reverse transcription (RT)–polymerase chain reaction (PCR) indicated that the gene expression levels of HO-1 remained unchanged in all groups; however, expression levels of inflammatory mediators, particularly interleukin (IL)-6 and IL-1β, were significantly higher in the air group than in the CO/O_2_ (IL-6: *p* = 0.015 and IL-1b: *p* < 0.001) and control (IL-6: *p* = 0.020 and IL-1b: *p* = 0.023) groups. In addition, there were no significant differences in expression levels of anti-inflammatory (transforming growth factor (TGF)-β, inducible nitric oxide synthase (iNOS), and granulocyte-macrophage colony-stimulating factor (GM-CSF)) and apoptotic (caspase 3) mediators (Figure 3).

### 2.2. Experiment 2. Comparison of Microscopic Findings and Arterial Blood Gas (ABG) of Canine Donor Lungs Ventilated From the Inside With Different Gases and Preserved in a High-Pressure CO/O_2_ Gas Mixture

**Figure 4:**
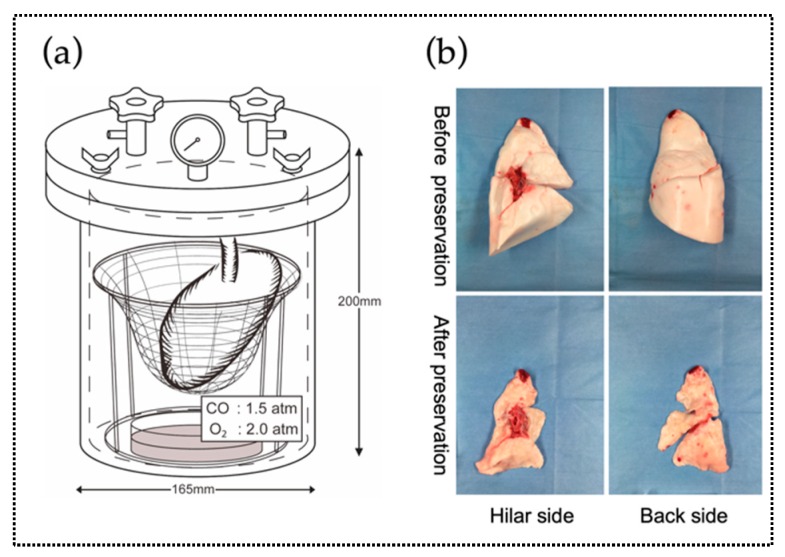
Schematic representation of the preservation method used in this study (Experiment 2). (**a**) The chamber was filled with a mixture of CO and O_2_ [PCO (partial carbon monoxide pressure), 1.5 atm; PO_2_ (partial oxygen pressure), 2 atm], and a flask with 50 mL distilled water was placed inside to maintain humidity for 20 h. The lung was gently placed on the net placed in the container. Before preservation, each lung was ventilated five times either with a mixture of CO and O_2_ at a ratio of 3:4 (CO/O_2_ group) or air (air group). (**b**) In the chamber, the trachea was kept open. After preservation, the lung shrunk due to high pressure.

Canine lung transplantation was compared between the CO/O_2_ and air groups. Since the canine lung was larger than the rat lung, a donor canine lung was placed on a net (Figure 4a). Before preservation, each lung was ventilated five times either with a mixture of CO and O_2_ at a ratio of 3:4 (CO/O_2_ group) or with air (air group). The size of the canine lungs after preservation reduced to about half of that before preservation because of the high pressure. (Figure 4b).

**Figure 5:**
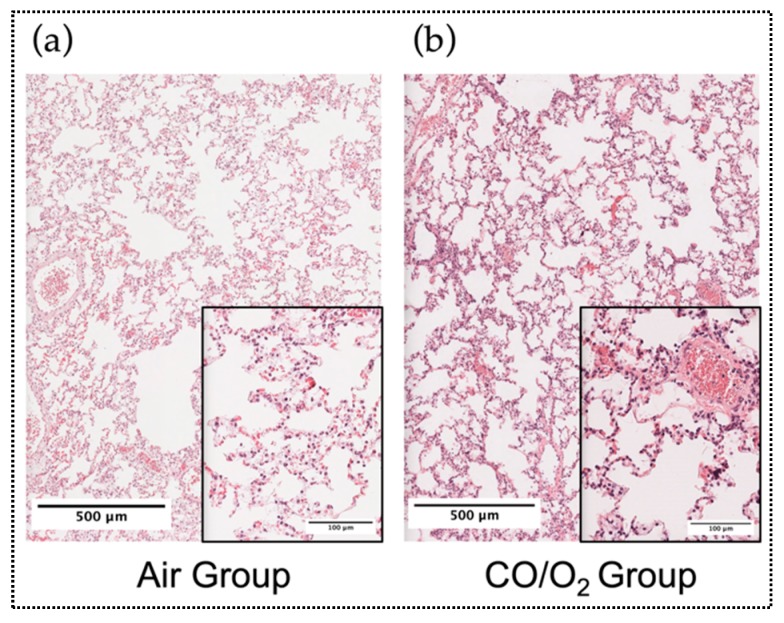
Light microscopic findings of the donor canine lungs after reperfusion by hematoxylin and eosin staining in the air (**a**) and CO/O_2_ groups (**b**).

Light microscopic evaluation revealed that the normal lung structure was well preserved in the air and CO/O_2_ groups (Figure 5a,b).

**Figure 6:**
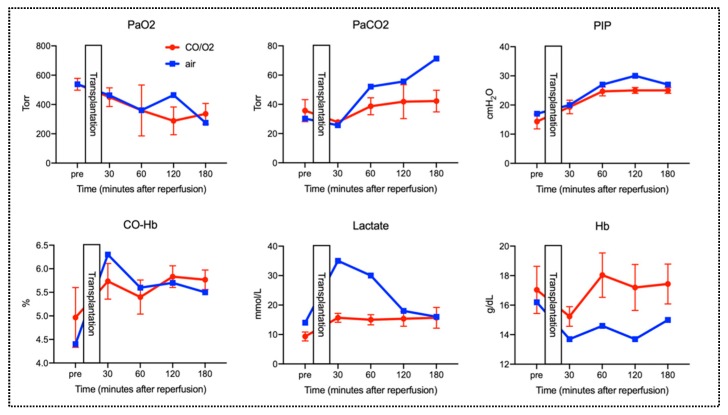
Assessment of chorological changes in parameters of arterial blood gases (ABG). The red line shows parameters in the CO/O_2_ group, and the blue line shows parameters in the air group. Each data of red lines are expressed as the means ± standard deviations.

**Figure 7:**
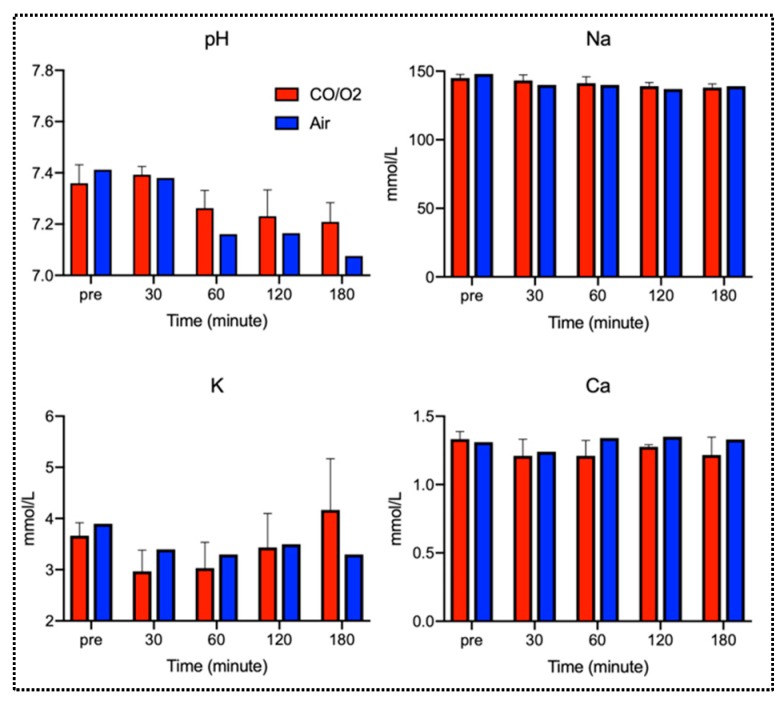
Arterial blood gas analysis for pH, Na, K, and Ca levels in the CO/O_2_ and air groups. The red bars show parameters in the CO/O_2_ group, and the blue bars show parameters in the air group. Each data of red bars are expressed as the means ± standard deviations.

Results of the arterial blood gas (ABG) assessment at multiple time points after reperfusion are presented in Figure 6. Briefly, there was no noticeable increase in CO-Hb concentration in any of the groups before or after reperfusion. Partial arterial oxygen pressure (PaO_2_) in the CO/O_2_ and air groups was 288.3 ± 94.7 and 464 torr, respectively, at 120 min after reperfusion and 336.3 ± 70.9 and 275 torr, respectively, at 180 min after reperfusion. These results indicated that a good O_2_ supply capacity after reperfusion was maintained in both the groups. In the CO/O_2_ group, the partial arterial carbon dioxide pressure (PaCO_2_) at 30, 60, 120, and 180 min after reperfusion was 35.7 ± 7.54, 27.9 ± 0.64, 41.8 ± 11.6, and 42.2 ± 11.6 torr, respectively. Conversely, PaCO_2_ at 30, 60, 120, and 180 min after reperfusion was 25.7, 52.1, 55.6, and 71.3 torr, respectively, in the air group, indicating the tendency for CO_2_ retention over time in the air group. Additionally, arterial blood lactate levels were high in the air group at 30 min after reperfusion. In both groups, peak inspiratory pressure (PIP) tended to increase starting at 60 min after reperfusion. PIP levels showed similar trends between the CO/O_2_ and air groups at other time points. In addition, other parameters (pH, Na, K, and Ca levels) showed similar trends between the CO/O_2_ and air groups at all time points (pre-operation, 30, 60, 120, and 180 min after reperfusion, Figure 7).

## 3. Discussion

This study demonstrated that filling rat lungs with a CO and O_2_ mixture via the HPG preservation method suppressed IRI. Furthermore, filling canine lungs with a CO and O_2_ mixture maintained PaO_2_ but suppressed the increase in lactic acid levels in the recipient.

Reportedly, HO-1 generates endogenous CO, and the HO-1/CO system confers tissue protective anti-inflammatory, anti-oxidative, and anti-apoptotic effects [20,21]. Recently, several trials have employed exogenous CO administration via inhalation or pharmacologic delivery [22,23,24]. However, the human body cannot be exposed to high concentrations of CO because it causes hypoxemia by generating CO-Hb [25]. Meanwhile, donor organs for transplantation may benefit from the effects of CO because they are preserved after removing blood, including hemoglobin (Hb), by the reflux of preservation solution. A previous study showed that rat heart transplantation using the HPG preservation method did not lead to increased CO-Hb concentration in recipients despite the exposure of the donor organs to high CO concentrations [17]. Similarly, the present study showed that CO-Hb concentration in the canine recipient remained unchanged until 180 min after reperfusion (Figure 6). These results indicate that the HPG preservation method, which does not affect the CO-Hb concentration in the recipient, is safe and could be one of the methods that can maximize the effects of the HO-1/CO system.

The lung, one of the most commonly transplanted organs, characteristically includes abundant alveolar spaces that are constantly exposed to gases, such as O_2_, N_2_, and CO_2_, during gas exchange [26]. Therefore, the lungs, unlike other organs, can be filled with gases, such as CO, both from the surface and from the inside in the HPG preservation method. This study demonstrated that the preserved rat lungs filled with air tended to be heavier and had more extensive and severe alveolar hemorrhage than those filled with the CO and O_2_ mixture when examined histologically. mRNA expression levels of HO-1 were not significantly different among the control, air, and CO/O_2_ groups. A previous study showed that exposure to exogenous CO increased HO-1 expression in the body [22]. However, this study suggested that exogenous CO administration during organ preservation using the HPG method did not activate the endogenous HO-1/CO system. Moreover, after reperfusion of the excised rat lungs, expression levels of various inflammatory mediators (IL-6 and IL-1β) were significantly increased in the lungs filled with air, but their levels were suppressed in the lungs filled with the CO and O_2_ mixture (Figure 3). Furthermore, western blotting was performed to assess IL-6 and IL-1β levels in the lungs. Similar to the results of real-time RT–PCR, IL-6 and IL-1β protein levels in the CO/O_2_ groups were lower than those in the air group (Figure S1).

These results indicate that exposure to the CO and O_2_ mixture from inside the lung is important and effective in reducing IRI during the HPG preservation method. Further examination revealed more severe alveolar hemorrhage in the lungs filled with CO or O_2_ alone than in the lungs filled with their mixture (Figure S2); therefore, both CO and O_2_ are essential. Further studies are warranted to determine more optimal gas combinations and ratios.

The characteristic structure of the lungs indicates the feasibility of the HPG preservation of large solid organs because the lungs can be filled with gases by delivery to every alveolus from the inside. Therefore, we examined the differences in the effects of HPG preservation based on organ size by comparing canine lungs, which are approximately half the size of adult human lungs, with rat lungs. Compared with the air group, the CO/O_2_ group demonstrated PaO_2_ preservation even after 180 min of reperfusion and exhibited a tendency of suppressed lactic acid production (Figure 5). These results indicate that the lungs may be one of the ideal organs that could benefit the greatest from the HPG preservation method because of their unique characteristics. We propose that adaptation of this method to human lungs, which have larger volumes, should be considered in the future.

Although the HPG preservation method can take full advantage of the effects of gases, pressure injury is one of the critical issues. To verify the pressure damage, we examined whether the trachea should be kept open when the lungs were exposed to the HPG mixture following their inflation. We found that there was considerable histological damage to the alveolar structure when the trachea was closed, which was evidently worse than that observed in the inflated lungs with the trachea kept open (Figure S3). Pressure trauma is a recognized injury that occurs in paranasal sinuses and the middle ear during diving and is believed to be due to a pressure gradient between the external environment and the closed lumen, resulting in relative negative pressure in the lumen and edema/bleeding of mucosal surfaces lining the lumen [27]. In the present study, there was a pressure gradient of 2.5 atm between the outside and inside of the lungs during HPG preservation in the group with the trachea closed at the time of preservation. Relative negative pressure on the alveolar tissue might have contributed, at least partially, to the alveolar damage. Therefore, to maximize the effect of inflation by gas for lung preservation while preventing pulmonary injury due to high pressure, the gas mixture should be delivered for lung inflation only before storage and the trachea should be kept open at the time of preservation.

The HPG preservation method used in this study can be applied to human lungs in a similar manner as that for canine lungs. However, during clinical lung transplantation, it is often necessary to transfer donor lungs from the donation institute to the transplantation institute; therefore, we think that some improvements in the storage chamber are necessary. First, because the chamber used in this study is not large enough to preserve the human lung, it is necessary to build a larger chamber. Second, the storage chamber should have a refrigeration function to perform cryopreservation, thereby preventing tissue necrosis during delivery. Finally, a small CO or O_2_ bomb should be attached in case CO or O_2_ needs to be refilled into the chamber during transport.

There are some possible limitations in this study. To focus on animal protection and minimize the number of experimental animals used, the efficacy of the HPG preservation method was first demonstrated in a technically simple rat ectopic transplant model. Second, the safety of this method was verified in an orthotopic transplant canine model with large organs. Therefore, the usefulness and safety of the method warrant evaluation in an animal model. Moreover, to protect animals, only one sample was included in the control group and no statistical analysis could thus be conducted in terms of the functional aspect of the donor lung in the canine model. To demonstrate the usefulness of the HPG preservation method for the lungs in large animals, additional experiments with canine model should be conducted. Finally, comparative studies with the existing simple immersion method were not conducted. Such a comparison is warranted prior to the clinical application the HPG preservation method in the future.

In conclusion, this study demonstrated that the physiological effects of the HO-1/CO system were employed for preserving donor lungs with unique characteristics via the HPG preservation method. This approach has significant potential to be used as a new preservation method for lungs.

## 4. Materials and Methods

This study was performed in accordance with the Guide for the Care and Use of Laboratory Animals prepared by the Institute of Laboratory Resources at the National Research Council (http://nap.edu/catalog/12910.html). In experiment 1, the Regulations for Experimental Animals, prepared by Aichi Medical University (2010), was followed after the approval of the study design by the Committee for Animal Experiments at Aichi Medical University (Authorization number: 2016-11, Authorization date: 3 June 2016). Similarly, experiment 2 followed the Rules on Animal Experiments in Kagawa University prepared by Kagawa University (2016) following the approval of the study design by the Committee for Animal Experiments at Kagawa University (Authorization number: 15105-01, Authorization date: 10 November 2015).

### 4.1. Experiment 1. Rat Lung Preservation for 24 h Using the HPG Preservation Method

#### 4.1.1. Animals

Ten-week-old male rats from the LEW/SsN Slc inbred line, with an average weight of 230 g (range, 220–245 g), were purchased from Shizuoka Laboratory Animal Center (Shizuoka, Japan). All rats were maintained under standard conditions and fed rodent food and water in accordance with the Regulations for Experimental Animals by Aichi Medical University (Authorization number: 2016-11).

#### 4.1.2. Anesthesia, Extraction, and Preservation of Donor Lungs, and Ectopic Lung Transplantation

Previous studies have achieved the long-term preservation of the rat heart using the HPG preservation method [15,16,17]. In the rat model used in this study, ectopic cervical lung transplantation was performed and IRI severity was evaluated according to previous studies.

The left lungs were extracted from rats under deep anesthesia using pentobarbital (50 mg/kg; Kyoritsu Seiyaku, Tokyo, Japan), and blood was removed by the organ preservation solution (ET-K solution, Otsuka Pharmaceutical, Tokyo, Japan) via retrograde reflux from the ascending aorta to the pulmonary artery. A custom-built 7 air pressure-resistant chamber (length, 165 mm; width, 165 mm; height, 200 mm; material, iron; Nakamura Iron Works, Tokyo, Japan) was cooled to 4 °C in advance. Before undergoing HPG preservation, donor lungs were filled with either room air (air group, *n* = 5) or a mixture of CO and O_2_ (CO/O_2_ group, *n* = 5). Rat lungs that were immediately transplanted after extraction were used as the control group (*n* = 4), whereas the lungs that were immediately analyzed after extraction were used as the normal group (*n* = 3). Next, the rat lungs were placed in the chamber filled with a CO and O_2_ mixture (3.5 atm; PCO, 1.5 atm, PO_2_, 2.0 atm) and contained a flask with 50 mL distilled water to maintain humidity for 24 h. Lung preservation was performed with the trachea kept open (Figure 1). After preservation, the lungs were removed from the chamber, and ectopic lung transplantation to recipient rats was performed with anastomoses of the pulmonary artery with the internal carotid artery and the pulmonary vein with the external jugular vein. Follow-up observation was performed for 90 min after the reperfusion, followed by excision of the transplanted lungs and weight measurement.

#### 4.1.3. Light Microscopy

Extracted rat lungs were processed with extended fixation in formalin solution for 24 h. The samples were washed, dehydrated in a series of graded ethanol, and embedded in paraffin. Serial sections (thickness, 6 µg) were cut using a microtome and stained with Gill’s hematoxylin III and 2% eosin Y. Tissue slides were digitized and stored using an image storage device (Aperio AT, Leika Biosystems, Wetzlar, Germany). The digitized slides were analyzed using imaging software (Aperio esilde manager, Leika Biosystems, Wetzlar, Germany). To assess IRI, alveolar hemorrhage rate was evaluated using for ImageJ (National Institutes of Health, Bethesda, MD, USA). For each tissue sample, five fields of view were randomly selected to be captured as images. The percent area occupied by red blood cells relative to the area of the entire image was defined as the alveolar hemorrhage rate.

#### 4.1.4. Analysis for Gene Expression Levels of Mediators

Gene expression levels of inflammatory mediators (TNF-α, IL-6, and IL-1β), anti-inflammatory mediators (HO-1, iNOS, and TGF-β), and apoptotic mediators (caspase 3 and GM-CSF) were quantified in duplicates using a two-step SYBR Green-based real-time RT–PCR protocol.

#### 4.1.5. Statistical Analysis

Values were presented as means and standard deviations. Significance in differences was determined using an analysis of variation (ANOVA) and post hoc tests. A *p* value of <0.05 was considered statistically significant.

### 4.2. Experiment 2. Canine Lung Preservation for 24 h Using the HPG Preservation Method

#### 4.2.1. Animals

Four pairs of weight-matched (8.9–11.0 kg) adult Narc Beagle canines (Kitayama Labes, Ina, Japan) were evaluated. All canines were maintained under standard conditions and fed dog chow and water in accordance with the Rules on Animal Experiments at Kagawa University (Authorization number: 15105-01).

#### 4.2.2. Anesthesia

All canines were anesthetized with intramuscular injection of ketamine (10 mg/kg; Daiichi Sankyo, Tokyo, Japan), xylazine (0.25 mg/kg; Bayer Yakuhin, Osaka, Japan), and atropine sulfate (0.05 mg/kg; Mitsubishi Tanabe Pharma, Osaka, Japan) and intubated for mechanical ventilation. Anesthesia was maintained via the inhalation of 2–3% sevoflurane (Pfizer, New York, NY, USA) and O_2_. All donor and recipient canines were ventilated with an inspired O_2_ fraction of 1.0 at a tidal volume of 20 mL/kg, a respiratory rate of 15 breaths/min, and a positive end-expiratory pressure of 5 cmH_2_O.

#### 4.2.3. Extraction of the Donor Lung and Preservation

We regarded the canine model as an intermediate stage for the clinical application of the HPG preservation method and emphasized the evaluation in a more clinical form. Therefore, the orthotopic transplantation of the donor lungs was performed for evaluation.

Catheters were introduced into the right femoral artery and the right external jugular vein. After the left thoracotomy, the left pulmonary artery and vein as well as the left main bronchus were encircled. After systemic heparinization (250 IU/kg; MOCHIDA PHARMACEUTICAL, Tokyo, Japan), bolus prostaglandin E1 (25 µg/kg; ONO PHARMACEUTICAL, Osaka, Japan) was injected into the right external jugular vein. After confirming that the atrial blood pressure (ABP) was at ≤90 mmHg, the left main pulmonary artery was cannulated with a 16-Fr-diameter catheter. After ligation of the proximal pulmonary artery and incision of the left atrium, the lung was flushed with 70 mL/kg ET-K solution to remove blood, with anterograde perfusion of the solution from the pulmonary artery and flushing with 30 cmH_2_O pressure. After perfusion, the left main bronchus and left atrium were incised, and the left lung was removed from inside the thoracic cavity. Donor lungs were preserved in a custom-built 7 air pressure-resistant chamber (Figure 4a).

#### 4.2.4. Allogeneic Lung Transplantation of the Preserved Lung and Functional Evaluation after Reperfusion

Recipient canines were anesthetized using the same approach for donor canines, and Swan-Ganz catheters were introduced to the right external jugular vein. Before surgery, heart rate, ABP, percutaneous O_2_ saturation, PIP, ABG, CO-binding hemoglobin (CO-Hb) concentration in blood, and systemic cardiac functions (pulmonary artery pressure, cardiac output, and pulmonary capillary wedge pressure) were evaluated. Left pneumonectomy was performed using the same approach for donor canines, and left single-lung transplantation was performed. Anastomosis was performed in the following order: Main bronchus, left atrium, and pulmonary artery. After 30 min of reperfusion, right main pulmonary artery and right main bronchus were ligated to evaluate the donor lung function. The ventilator settings were changed to achieve 2/3 of the tidal volume before reperfusion and a respiratory rate of 20 breaths/min. Heart rate, ABP, percutaneous O_2_ saturation, and PIP were measured at five time points: Pre-operation and 30, 60, 120, and 180 min after reperfusion. After 180 min of reperfusion, the transplanted lungs were excised for histological evaluation.

#### 4.2.5. Assessment of ABG and CO-Hb Concentration

Arterial blood obtained from the recipient canines at the indicated five time points were analyzed to measure partial arterial O_2_ pressure (PaO_2_), partial arterial CO_2_ pressure (PaCO_2_), CO-Hb concentration, lactate, Na, K, and Ca using a radiometer (ABL800 FLEX blood gas analyzer, Copenhagen, Denmark). Furthermore, ABG data mentioned above were considered to be more accurate because the absence of any increase in CO-Hb concentration was reflected in the minimal effect on the PaO_2_ value by ABG analysis.

#### 4.2.6. Light Microscopy

Canine lungs were processed by extended fixation in 10% formalin solution for 24 h. The samples were washed, dehydrated in a series of graded ethanol, and embedded in paraffin. Serial sections (thickness, 6 µm) were cut with a microtome and stained with Gill’s hematoxylin III and 2% eosin Y. Tissue slides were digitized and stored using an image storage device (Aperio AT, Leika Biosystems, Wetzlar, Germany). The digitized slides were analyzed using imaging software (Aperio esilde manager, Leika Biosystems, Wetzlar, Germany).

#### 4.2.7. Statistical Analysis

Values are presented as means and standard deviation.

## Supplementary Materials

Supplementary materials can be found at https://www.mdpi.com/1422-0067/20/11/2719/s1.

## Abbreviations

CO	Carbon monoxide
CO-Hb	Carboxyhemoglobin
HO-1	Heme oxygenase-1
MAPK	Mitogen-activated protein kinase
IRI	Ischemia–reperfusion injury
HPG	High-pressure gas
O_2_	Oxygen
N_2_	Nitrogen
RT–PCR	Reverse transcription–polymerase chain reaction
IL	Interleukin
TGF	Transforming growth factor
iNOS	Inducible nitric oxide synthase
GM-CSF	Granulocyte-macrophage colony-stimulating factor
ABG	Arterial blood gas
PaO_2_	Partial arterial oxygen pressure
PaCO_2_	Partial arterial carbon dioxide pressure
PCO	Partial carbon monoxide pressure
PO_2_	Partial oxygen pressure
CO_2_	Carbon dioxide
PIP	Peak inspiratory pressure
Hb	Hemoglobin
TNF	Tumor necrosis factor
ANOVA	Analysis of variation
ABP	Arterial blood pressure
RIPA	Radioimmunoprecipitation assay
SDS-PAGE	SDS-polyacrylamide gel electrophoresis
PVDF	Polyvinylidene di fluoride
