# Supplementary material for: High-Pressure Carbon Monoxide and Oxygen Mixture is Effective for Lung Preservation
Source: Int J Mol Sci. 2019 Jun 3;20(11):2719. doi: 10.3390/ijms20112719 (PMC6600409; doi:10.3390/ijms20112719)
Supplement: Supplementary file 1 [file ijms-20-02719-s001.pdf]

International Journal of
Molecular Sciences

MDPI

Supplementary Materials:

Supplemental experiment 1. Assessment of IL-6 and IL-1β expressions in donor lung tissues using western blotting.

We extracted proteins from rat lung tissues using a radioimmunoprecipitation assay (RIPA) lysis buffer. SDS-polyacrylamide gel electrophoresis (SDS-PAGE) was performed, and bands were transferred to polyvinylidene difluoride (PVDF) membranes. After blocking with 5% skim milk, antibodies against IL-6 (Rabbit anti-rat, #ab9324, Abcam, United Kingdom) and IL-1β (Mouse anti-rat, #ab9787, Abcam, United Kingdom) were added to analyze protein expression. Consequently, the membranes were incubated with the corresponding secondary antibodies (Peroxidase-Conjugated Goat anti-Rabbit IgG, #A0545, Sigma, United Kingdom and Peroxidase-Conjugated Goat anti-Mouse IgG, #115-035-062, Jackson, United States). The gray intensity of the band was evaluated using Image J (National Institutes of Health, USA). As an indicator of protein expression, the band gray intensity ratio of β-actin to IL-6 or IL-1b was calculated and evaluated. As with experiment 1, western blotting results were compared among the four groups.

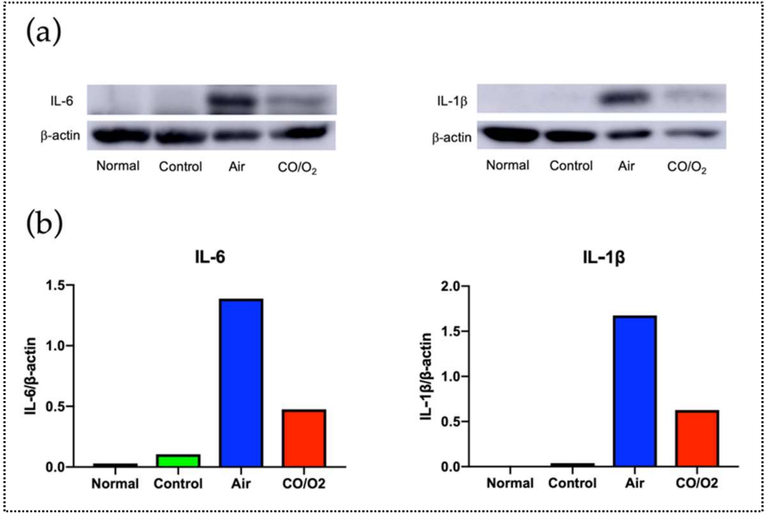

Figure S1. Evaluation of expression of inflammatory cytokines in rat donor lung tissues. (**a**) Protein expression using western blotting. (**b**) Comparison of the band gray intensity ratios of β-actin to IL-6 or IL-1b among the four groups.

Similar to the results of real-time RT–PCR results, IL-6 and IL-1β protein expression levels were lower in the CO/O2 groups than in the air group (Supplemental Experiment 1).

Supplemental experiment 2. Determination of the most appropriate gas mixture for the HPG preservation of rat lungs.

The appropriate gas mixture for lung inflation before its preservation was determined. The donor lung was excised as mentioned in the methods section and flushed with the ET-K solution. Ventilation was performed inside the lung with one of the following gas mixtures: 100% CO (n = 3), 100% O_2 (n = 3), or CO/O_2 (n = 5). After ventilation, the trachea was kept open and the lung preservation was performed for 24 h. Next, the donor lung was ectopically transplanted, followed by 90 min of reperfusion. The donor lung was then excised, and histological evaluation was performed to evaluate alveolar hemorrhage, as described in the methods section.

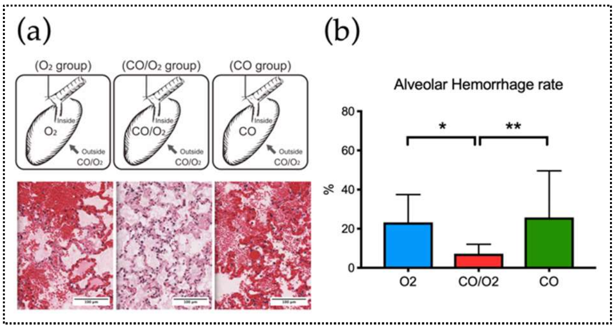

Figure S2. Evaluation of different gases for their utility in preservation. (**a**) Schematic representation of the experimental design and light microscopic findings in tissue samples from donor lungs of the three groups stained with hematoxylin and eosin. (**b**) Histological assessment for alveolar hemorrhage in the three groups. Each data of bars are expressed as the means ± standard deviations. * $p < 0.05$, * $p < 0.01$. Black bar = 100μm

Light microscopic evaluation performed in the CO, O_2, and CO/O_2 groups revealed that the degree of alveolar hemorrhage was significantly milder in the CO/O_2 group than in the O_2 and CO groups (Supplemental Figure 2).

Supplemental experiment 3. Determination of the tracheal position (close versus open) before the HPG preservation of rat lungs using CO/O_2.

Whether the trachea should be open during lung preservation was examined in the hyperbaric chamber. As described in the methods section, the donor lung was excised and flushed with the ET-K solution. Ventilation was performed inside the lung with the CO and O_2 mixture. Thereafter, the lung was preserved in one of the following conditions: Trachea open ($n = 5$) and trachea closed with the lung inflated ($n = 2$). Lung preservation was performed for 24 h. After preservation, as described in the methods section, the donor lung was ectopically transplanted and reperfused for 90 min. The donor lung was excised, and histological evaluation was performed to evaluate alveolar hemorrhage, as described in the methods section.

Light microscopic evaluation of preservation following the open- or close-trachea approach revealed that the alveolar hemorrhage was more severe in the close-trachea group than in the open-trachea group (Supplemental Figure 3).

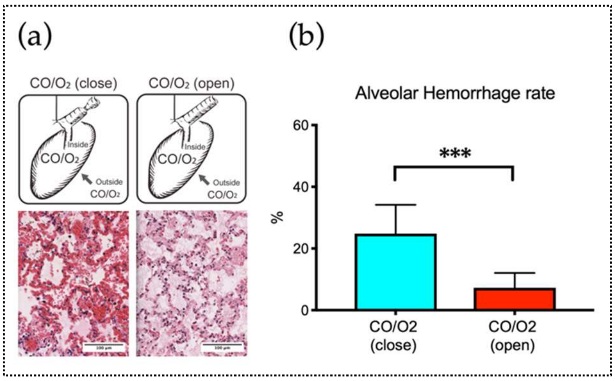

Figure S3. Evaluation of the tracheal position (close versus open) before the preservation of rat lungs using CO/O_2. (**a**) Schematic representation of the experimental design and light microscopic findings of donor lungs. (**b**) Histological assessment for alveolar hemorrhage. Each data of bars are expressed as the means ± standard deviations. ***$p < 0.001$. Black bar = 100μm

Table S1. Target gene names using real-time reverse transcription (RT)–polymerase chain reaction (PCR), accession numbers, and specific primer pair sequences.

Name	Accession Number	Direction	Sequence 5′ to 3′
TNF-α	NM_012675.3	Forward	tgtgcctcagcctcttctc
		Reverse	gagcccatttgggaacttct
IL-6	NM_012589.1	Forward	cccttcaggaacagctatgaa
		Reverse	acaacatcagtcccaagaagg
IL-1β	NM_031512.2	Forward	tgtgatgaaagacggcacac
		Reverse	cttcttctttgggtattgtttgg
TGF-β	AY_550025.1	Forward	cctggaaagggctcaacac
		Reverse	cagttcttctctgtggagctga
iNOS	NM_012580	Forward	cccagagtctctagacctcaaca
		Reverse	catggtgaacacgttcttgg
HO-1	NM_012580	Forward	gtcaagcacagggtgacaga
		Reverse	ctgcagctcctcaaacagc
GM-CSF	U_00620	Forward	catctctaatgagttctccatccag
		Reverse	cccgtagaccctgcttgtat
Caspase-3	NM_012922.2	Forward	ccgacttcctgtatgcttactcta
		Reverse	catgacccgtcccttcaa
